# Injury prevention: Individual factors affecting adult recreational snowboarders’ actual and estimated speeds on regular slopes

**DOI:** 10.1371/journal.pone.0246931

**Published:** 2021-02-10

**Authors:** Luis Carus, Isabel Castillo

**Affiliations:** 1 Department of Business Management, University of Zaragoza, Huesca, Aragón, Spain; 2 Department of Statistical Methods, University of Zaragoza, Huesca, Aragón, Spain; Al Mansour University College-Baghdad-Iraq, IRAQ

## Abstract

Speed is a main factor affecting the kinematic of snow-sports accidents and the degree of severity of the resulting injuries. The aim of this study was to measure on-slope actual maximum speeds of snowboarders and to assess their ability to accurately them with regard to individual factors such as gender, skill level, age and risk-taking behaviour and actual maximum speed. The data were obtained from a sample of 312 (67% male, 33% female) adult recreational snowboarders taking lessons in one of the major resorts in the Spanish Pyrenees. The Pearson correlation coefficient was used to investigate the relationship between maximal measured actual speed and estimated speed for all participants. Multiple linear regression analysis was used to estimate the impact of individual factors on both the snowboarders’ actual maximum speed and their error of estimation. The Pearson correlation coefficient between estimated and actual maximum speed was 0.52 (P < 0.001) for all participants. They underestimated their actual maximum speed on average by 10.05 km/h or 28.62%. All assessed factors were shown to significantly affect the snowboarders’ actual maximum speed. However, gender, skill level, age and actual maximum speed were shown to significantly affect the snowboarders’ error of estimation, while risk-taking behavior did not. Gender, skill level, age and risk-taking behaviour are associated with the actual maximum speed at which snowboarders ride, while the same individual factors, except for risk-taking behaviour, and their snowboarding speed seem to affect the ability to estimate actual maximum speeds in adult recreational snowboarders. The ability to estimate actual speed accurately is an important factor to avoid accidents on ski slopes and, therefore, having snowboarders informed about the benefits of speed self-awareness is a key matter for prevention purposes.

## Introduction

Injury prevention in sports facilities has been defined as the general planning, human and material resources allocation, and implementation and control of all measures needed to minimize the risk of accident occurrence and the severity of resulting injuries in sport facilities [1]. Undoubtedly, snow sports entail the risks of accident and injury, attributable either to oneself or to third parties, leading to more or less severe and undesirable ethical, legal and economic effects, which impel ski resorts to take the necessary steps to minimize the risk of their occurrence [2, 3].

One of the main factors affecting the occurrence of on-slope accidents and the severity of any resulting injury in ski resorts is the speed of the actors prior to the accident [4, 5]. Past research has shown how ski and snowboard accidents on regular slopes are very often the result of excessive speed and how the severity of the resulting injuries is likely to increase as speed increases, to the extent that fatalities are very often the result of high-speed accidents [4–9]. The origin of this connection between speed, accidents and the severity of resulting injuries can be found in the fact that for a given mass, kinetic energy increases as the square of the velocity (KE = ½ mv^2^), and that high speed negatively affects the time and distance needed to conduct an adequate response to avoid obstacles or other persons [10, 11].

It follows that to prevent speed-related injuries in ski resorts, preventive strategies must include avoiding excessive speed during skiing and snowboarding, as different alpine responsibility codes (issued by ski resorts, winter sports national federations, clubs, etc.) and the International Ski Federation (FIS) rules for conduct on slopes have recommended–since they were first issued in 1967, though there has been a subsequent update to include snowboarders in 2002– [12, 13]. Consequently, in addition to other typical speed control strategies undertaken by ski resorts, such as using posted speed recommendations or limits, ski patrollers enforcing rules against high-speed, or creating properly balanced fenced areas and mazes with the intention of requiring a safe reduction in speed [3, 14], as Ruedl et al. [15] put it for the case of skiers, would be advantageous if users could estimate their individual speed as accurately as possible. However, as Dickson et al. [3] remark, given that increasing skill levels in many sports are linked to greater speeds, the concern is that new devices designed to inform users of their actual speed will add risk as risk-seeking participants strive to better themselves in terms of how fast they go.

Furthermore, for them to devise excessive speed effective prevention policies, it would be advantageous that ski resorts’ managers counted on useful insights on the factors that affect the users’ actual speed and their ability to estimate it. As Dickson et al. [3] recap, to be effective at controlling speed, there must be an understanding of the speed at which participants are moving, both by the participants themselves and by those seeking to modify or manage the participants’ behaviour. Besides, a sound understanding of snowboarding speed is also useful to assess protective equipment such as helmets used for recreational snow sports, bindings or ski area padding whose effectiveness depends on the impact speed and the energy involved in the accident [15–17].

To that ends, previous studies have consistently analyzed how actual speed of on-slopes snow-sports participants is related to various factors [3, 15, 16, 18–20]. Some of these studies have studied the skiers’ ability to qualitatively perceive their actual speed [15, 16], others have studied the skiers’ ability to quantitatively estimate it [3, 15, 18–20], and one studied such ability among a small subset of a quantitatively indiscriminate mixture of skiers and snowboarders [19]. However, to the best of our knowledge, no study as of yet has been specifically devoted to evaluating the actual speeds of the snowboarders and their ability to estimate them.

Therefore, given the importance of the subject for injury prevention policies design and for the assessment of protective equipment, the aim of this study was to analyse the actual maximum speeds of snowboarders on groomed slopes with regard to gender, skill level, age and risk-taking behaviour, and their ability to accurately estimate it with regard to the aforementioned factors and their actual maximum speed.

## Materials and methods

This study was conducted following the approval of the University of Zaragoza research board, and the research was carried out following the rules of the Declaration of Helsinki, regarding research involving human subjects (revised in 2013 in Fortaleza, Brazil).

Data for this study were collected between December 2019 and mid-March 2020 at a major ski resort in the Spanish Pyrenees, from a sample of 312 adult recreational snowboarders taking snowboard lessons. All of them gave their informed consent to participate in the experiment.

The inclusion criterion was an age of 18 years or older. Participants were chosen to largely represent gender, skill level and age groups in similar proportions to those of the ski school’s usual populations of snowboarders. Data on gender and age–classified into four groups (≤30, 31–40, 41–50, >50) [16, 18]–were recorded at the time of customer registration. However, when the statistical analysis was first applied to the original data under the consideration of the four age groups, it revealed that only the group of snowboarders over 50 years had shown significantly different actual maximum speeds versus the other three groups, which showed no significant differences among them. For that reason, age was recoded to conform to two groups of age (>50 vs ≤50).

With regard to skill levels, it is a ski school mandatory procedure that all customers taking lessons demonstrate their maximum skill under the observation of Level III–certified instructors, who then decide about their skill level on the spot according to Sulheim et al. [21]. Once skill levels had been so established were then grouped into less-skilled (complete beginners and low levels) and more-skilled (advanced and experts) [16, 18]. Helmets were mandatory for all participants in accordance with the ski school’s rules.

Regarding risk-taking behaviour, in this study it is understood to be associated with the personality trait sensation seeking in reference to the seeking of intense sensations and experiences, and the willingness to take physical and social risk for the sake of such experience [22].

Speed measurements were obtained on groomed runs because previous research has shown that most snow sports injuries and fatalities occur on well prepared slopes [23, 24], and because ungroomed runs tend not to lend themselves to fast skiing. These trails are more likely to be exceptionally steep, narrow, bumpy and twisty; thus, speeds attained on them tend not to be as fast as groomed trails [3, 19, 23].

Speed measurements were gathered in different environmental conditions and at different times by a team of experienced snowboard instructors in their normal day’s instructional activity [25]. Each was furnished with a smartphone preloaded with a recent (December, 2019) version of a GPS-based snow-sports application for Android (Ski Tracks 1.3.17; Core Coders Ltd.), previously and satisfactorily used in snow sports speed measurements and which, among other variables (altitude, number of runs, slope inclination, route followed, etc.), could record maximum actual speeds [25]. We had its precision previously validated by comparing Ski Tracks’ results with photocells measurements on a closed racing stadium; the results proved negligible mean time differences with no systematic bias [26].

Instructors received specific instruction on the operation of the application and were made aware that participants might attempt to ride at speeds higher than their normal ones due to being measured; therefore, they stressed to participants that the research focused on normal snowboarding of the mountain (rhythm, line or combination of turns size) and to put as little emphasis as possible on the measurement of speed, though participants were aware that this measurement was taking place [3].

At the starting point of a run matching the participants’ skill level and after the data-recording devices had been fitted, snowboarders were asked to keep them on all the way through the completion of a whole run at their will and ease, in the same way they would do if snowboarding on their own. At the bottom of the hill they were asked to estimate what their maximum speed had been and whether they thought themselves to be cautious or risk-taking snowboarders [22].

Positive (overestimation) and negative (underestimation) errors of estimation (EE) were the resulting differences between estimated speeds, as perceived by the participants, and actual speeds, as recorded by the apparatus. The closer the EE is to 0, the better was the snowboarders’ ability to estimate his or her speed.

Participants’ data were entered into Minitab 19 for Windows (State College, PA, USA) for analysis and included descriptive statistics to summarize the data (e.g. mean and standard deviation, as well as maximum speeds) and between-group differences.

The Pearson correlation coefficient and the intraclass correlation coefficients (ICC) were used to investigate the relationship between actual maximum speed and estimated speed for all participants [3, 15, 18, 19, 25]. Multiple linear regression analysis was used to estimate the effect of individual characteristics on both the snowboarders’ actual maximum speed and their EE. All probability values were two-tailed, and values of .05 or less were considered to indicate statistical significance.

## Results

A total of 312 adult snowboarders (209 [67%] male, 103 [33%] female) with a mean (±SD) age of 35.5 (±10.4) years participated in this study.

Mean actual maximum speed and mean estimated speeds of all participants were 43.33 (±14.07) km/h and 33.29 (±18.48) km/h, respectively. The Pearson correlation coefficient between the actual maximum speeds and the estimated speeds was 0.54 (P < 0.001) for all participants, while the intraclass correlation coefficient was 0.77 (P = 0.029). A 95.2% (297) of them underestimated their actual maximum speed while a 4.8% (15) overestimated it. Participants’ median absolute error of estimation (MAE) was 10.23 (±6.47) km/h, while they underestimated their actual speed on average by 10.05 (±6.75) km/h (S1 Fig).

The maximum actual speed was 72.7 km/h, for a male, more-skilled, risky, and between 31–40 years of age, while the minimum was 7.1 km/h, for a female, less-skilled, cautious, and older than 50 years of age. The highest error was an underestimation of 25.6 km/h or 71.91% of the actual maximum speed, while the lowest was an overestimation of 3.2 km/h (5.63%).

Mean actual maximum snowboarding speed and mean EEs with regard to gender, skill level, age and risk-taking behaviour are displayed in Table 1.

**Table 1:** Participants profile and mean values (±SD) of actual maximum speed and errors of estimation with regard to gender, skill level, age and risk-taking behaviour.

Factors	n (%)	Actual maximum speed (km/h)	EE (km/h)		
			Underestimations 297 (95.2%)	Overestimations 15 (4.8%)	Sample* 312 (100%)
Gender					
Male	209 (67)	46.35 (±13.60)	-9.85 (±6.41)	2.24 (±0.88)	-9.27 (±6.77)
Female	103 (33)	37.22 (±12.97)	-12.29 (±5.86)	1.32 (±0.73)	-11.63 (±6.43)
Skill level					
More-skilled	144 (46)	57.12 (±6.28)	-6.36 (±4.60)	1.93 (±0.94)	-5.49 (±5.04)
Less-skilled	168 (54)	31.51 (±5.56)	-13.95 (±5.46)	-----	-13.95 (±5.46)
Age					
≤50	265 (85)	43.83 (±14.26)	-10.32 (±6.25)	1.90 (±0.96)	-9.68 (±6.67)
>50	47 (15)	40.54 (±12.53)	-12.45 (±6.48)	2.35 (±0.05)	-12.14 (±6.83)
Risk-taking behavior					
Risky	110 (35)	49.97 (±13.85)	-13.48 (±7.31)	1.95 (±0.25)	-13.34 (±7.44)
Cautious	202 (65)	39.72 (±12.81)	-9.01 (±5.02)	1.88 (±0.95)	-8.26 (±5.58)
Total	312	43.33 (±14.07)	-10.65 (±6.34)	1.93 (±0.94)	-10.05 (±6.75)
					10.23 (±6.47)

* Negative values show an overall underestimation of actual maximum speeds. The positive value shows the median absolute error (MAE) of estimation of actual maximum speeds.

-----None of the less-skilled snowboarders overestimated their actual maximum speed.

Regarding actual maximum speed, the multiple linear regression analysis, with entering all factors [gender, skill level, age and risk-taking behaviour], showed a significant impact of all of them on actual maximum snowboarding speed (Table 2).

**Table 2:** Results of the multiple linear regression analysis of factors affecting the actual maximum speed.

Factor	B^a^	SE B^b^	t	P value
Constant	64.93	.18	353.86	<0.001
Male* vs female	-8.06	.19	-42.16	<0.001
More-skilled* vs less-skilled	-25.20	.18	-140.59	<0.001
≤50* vs >50	-5.30	.25	-21.07	<0.001
Risky* vs cautious	-7.05	.19	-37.23	<0.001

^a^ Unstandardized coefficient.

^b^ Standard error of B.

* Reference category.

The final model of the multiple linear regression analysis explains up to 98% of the variance of actual speed. In order to account for high R and R^2^ values cross-validation was performed but differences were only obtained in the third and fourth units after de decimal point. Actually, the values of the coefficient of determination barely changed, what led us to believe that the model is dependable enough to make predictions (Table 3).

**Table 3:** Model summary*.

Model	R	R^2^	Adjusted R^2^	R^2^ change	R^2^ pred	Significant chance	Durbin-Watson
I^a^	0.30	0.09	0.0903	0.09	0.0817	.000	0.07
II^b^	0.95	0.90	0.9052	0.81	0.9040	.000	0.65
III^c^	0.96	0.93	0.9316	0.03	0.9306	.000	0.68
IV^d^	0.99	0.98	0.9876	0.05	0.9873	.000	0.73

* Dependent variable: actual maximum speed in km/h.

^a^ Predictors: gender.

^b^ Predictors: gender, skill level.

^c^ Predictors: gender, skill level, age.

^d^ Predictors: gender, skill level, age, risk-taking behavior.

The results of the multiple linear regression analysis (Table 2) reveal that men who were more-skilled and rated themselves as risky snowboarders and those younger than 50 years rode significantly faster by a mean of 8, 25.2, 7 and 5.3 km/h compared to women who were less-skilled, more cautious, and those older than 50 years, respectively.

With regard to the EE, the results of the multiple linear regression analysis are shown in Table 4. They show a significant impact of the actual speed, gender, skill level and age on the participants’ ability to estimate their actual maximum speed, but not of self-rated risk-taking behaviour.

**Table 4:** Results of the multiple hierarchical regression analysis of factors affecting the EE.

Factor	B^a^	SE B^b^	t	P value
Constant	37.04	9.19	4.03	<0.001
Actual speed	-0.68	0.14	-4.87	<0.001
Male* vs female	-8.79	1.24	-7.11	<0.001
More-skilled* vs less-skilled	-26.37	3.59	-7.34	<0.001
≤50* vs >50	-8.12	0.97	-8.33	<0.001
Risky* vs cautious	1.62	1.10	1.47	0.14

^a^ Unstandardized coefficient.

^b^ Standard error of B.

* Reference category.

Even though self-rated risk-taking behaviour does not show statistical significance, previous research by Ruedl et al. [18] showed an impac of this factor on the ability of skiers to estimate speeds as accurately as possible. Therefore, all factors were considered in the final model. The final model of the multiple linear regression analysis explained 67% of the variance of the EE (Table 5).

**Table 5:** Model summary*.

Model	R	R^2^	Adjusted R^2^	R^2^ change	Significant chance	Durbin-Watson
I^a^	0.50	0.26	0.26	0.26	.000	1.24
II^b^	0.50	0.26	0.26	0.00	.088	1.24
III^c^	0.71	0.51	0.50	0.25	.000	1.56
IV^d^	0.81	0.67	0.67	0.16	.000	1.88
V^e^	0.82	0.67	0.67	0.00	.142	1.89

* Dependent variable: error of estimation (EE) in km/h.

^a^ Predictors: actual speed (km/h).

^b^ Predictors: actual speed (km/h), gender.

^c^ Predictors: actual speed (km/h), gender, skill level.

^d^ Predictors: actual speed (km/h), gender, skill level, age.

^e^ Predictors: actual speed (km/h), gender, skill level, age, risk-taking behavior.

The results of the multiple linear regression analysis (Table 4) reveal that when snowboarding speed increased 1 km/h, the EE significantly decreased by 0.68 km/h. Women, less-skilled snowboarders and snowboarders older than 50 years estimated their actual maximum speed less accurately by a mean of 8.8, 26.4 and 8.1 km/h compared to men who were more-skilled snowboarders and snowboarders of or younger than 50 years of age, respectively.

## Discussion

Gaining insight into snowboarding speed on regular slopes is decisive to devise successful injury prevention policies and to evaluate means for users’ protection. Consequently, this research aims at delving into the understanding of snowboarding speed by studying how individual personal factors influence adult recreational snowboarders’ speed and their ability to estimate it, the intention being that the results obtained will help snowboarders, resort managers and politicians to make informed decisions about managing on-slope behaviours for the safety of all snowboarders. For this purpose, the study benefited from using GPS-based technology, which has been showed to be adequate for snow-sports speed measurements [26, 27]. It consisted of a smartphone new GPS-based application that provided advantages in terms of flexibility, affordability, and simplicity of operation or public accessibility compared to the radar guns that were previously used for measuring on-slope speed [25].

In general terms, the main results of this research were that all the individual factors under study seem to affect the actual speed of recreational adult snowboarders, and that snowboarding actual speed, gender, skill level and age seem to affect snowboarders’ ability to estimate their actual maximum speeds, whereas risk-taking behaviour does not. The reported Pearson product-moment correlation coefficient between the actual and estimated maximum speeds obtained for snowboarders in this study is similar to that obtained for skiers in previous studies [15, 18, 19]. However, regarding actual speed, the mean speed obtained in this study for snowboarders of all skill levels is lower than any of those previously measured for skiers [15, 18, 19]. This means that for skiers and snowboarders of equal mass, the average skier has more kinetic energy (KE = ½ mv^2^) than does the average snowboarder.

In comparison to our results, previous research using radar speed guns by Shealy et al. [19] and Bailly et al. [15] reported an average of maximum speed recorded for snowboarders of ~39 km/h, which is lower than that obtained in this study by ~4 km/h. or 2.5%. However, the radar guns they used only measured subjects moving in line with the radar beam, which was not always the case of the subject because of the turns performed, and might have led to an underestimation of the actual speed [15]. Therefore, that difference might be accounted for by the different devices used for measuring purposes and by differences in the sample composition with respect to gender, skill level, age and risk-taking behavior.

With respect to the skill level, the difference of 25.61 km/h between the average actual maximum speed of less-skilled and more-skilled snowboarders was the greatest difference noted for any of the predictors, and translates, for a given mass, into kinetic energy increasing about 3.3 times. Our results show that males rode faster than females, what might be attributable to an on average higher skill level and more risky behavior in men [18, 28, 29]. Male snowboarders rode on average at 46.35 km/h, versus only 37.22 km/h for females. This average speed difference translates into 54% greater kinetic energy for male snowboarders compared to female snowboarders for snowboarders of equal mass. However, because adult males weigh more than adult females, on average male snowboarders will have even more kinetic energy. For example, assuming that the average adult male weighs ~77 kg and the average adult female ~62, an average adult male snowboarder at 46.35 km/h will have ~92% more kinetic energy than an average adult female snowboarder at 37.22 km/h. Risky snowboarders rode on average at 49.97 km/h, versus only 39.72 km/h for the cautious. These average speed difference translate into 1.5 greater kinetic energy for risky snowboarders compared to cautious snowboarders, for snowboarders of equal mass. In comparison to those older than 50 years, younger snowboarders rode on average significantly faster, which might be due to a better physical fitness level and a riskier behavior in younger ages [22, 28].

With regard to speed estimation, our finding that actual maximum speeds were underestimated on average by 10.05 km/h is similar to the observations of Shealy et al. [19] for a quantitatively undetermined mixture of snowboarders and skiers. In contrast to previous research by Bailly et al. [15] and Ruedl et al. [18], who encountered skiers totally unable to estimate their actual maximum speed by percentages lower than 240% and 300%, respectively, in this study snowboarders never made errors of estimation exceeding 72%. Similar to what has been shown to be the case with skiers [18], our results suggest that the ability to estimate speed was affected by the snowboarder’s actual speed. However, in contrast to skiers, snowboarders almost always (95.2%) underestimated their maximum speed and, in alignment with Shealy et al. [19], they tended to increasingly underestimate their speed the faster they went.

Also, similar to the case of skiers [18], besides snowboarders’ speed, mainly skill followed by gender and age seemed to significantly impact on the ability to estimate maximum speeds as accurately as possible. However, different from skiers, the risk-taking behaviour did not. Men, more-skilled snowboarders and snowboarders of or younger than 50 years of age showed a significantly better ability to estimate their actual maximum speed compared to women, less-skilled snowboarders and snowboarders older than 50 years, respectively. In this sense, we could speculate, as Ruedl et al. [18] did regarding skiers, that more-skilled snowboarders, mostly men, ride more days per season and have more snowboarding experience compared to less-skilled snowboarders, which results in a better ability to accurately estimate their speed, and that women showing on average a lower ability to estimate their speeds might be partly related to the greater proportion of less-skilled snowboarders among them found in this study. In comparison to those who were younger, snowboarders older than 50 showed a worse ability to estimate speed, which might be due to an age-related decline in the ability to sense changes in velocity [30].

Even when snowboarders rode at the lowest mean speed (31.51 km/h for less-skilled), their speed was above the ASTM F2040 “Standard Specification for Helmets Used for Recreational Snow Sports” evaluation criterion of 22.3 km/h. (2.0 m drop test onto a flat steel anvil steel with a 300 g max acceleration limit) [31]. Compared to this benchmark, up to 97% of the participants’ maximum speeds were higher than that set by the standard, which means that the kinetic energy of a snowboarder riding within the range of the lowest and the highest mean actual speeds (57.12 km/h for more-skilled) is between 1.4 and 2.5 times greater than at 22.6 km/h. However, regarding helmet effectiveness in snowboarding the former reflection must be read with caution because, though helmets offer a finite amount of head protection [32], they may protect against impacts at higher speeds than what is found in the standards because these apply linear impact tests and do not address other factors that may concur in an accident, such as rotational forces, effective mass at impact, impact angle, type of object, quality of snow surface, etc. [17, 33, 34]. As Dickson et al. [3] put it, at a time when resort managers and legislators are increasingly considering making the wearing of helmets by resort guests mandatory, and snow sports schools, like the one that cooperated in this study, are making helmets a compulsory part of their instructors’ and guests’ gear, this highlights the need to further investigate the effectiveness of current snow-sports helmets designs to better meet the needs of current snow-sport behaviours.

Our findings suggest that information on snowboarders’ speeds and factors affecting their ability to estimate them must be a matter of interest to resort managers and politicians in the development of their injury prevention strategies. They could identify actual and estimated speeds associated with different groups of snowboarders and, together with a thorough understanding of usage patterns across the resort, might assist them with trail management, signage, and the implementation of safety strategies for areas of high use and where high-speed trails merge with areas used by less experienced participants [27].

On the other hand, the conduct of people participating in snow sports highly depends on their level of knowledge regarding the existing rules [35], and a decisive way of reducing accidents and the severity of resulting injuries related to speed would be to ensure that they are sufficiently familiar with the fact that they must adapt their speed and manner of riding to their ability and to the prevailing environmental conditions as well as to the density of traffic on the ski slope [13, 36].

This study evinces that snowboarders in general have a very poor idea of how fast they ride. It seems necessary that ski resorts undertake policies meant to inform users on how the odds of having an accident and the severity of resulting injuries increase with speed and about the benefits of speed self-awareness for prevention purposes. According to Ruedl et al. [18], the ability to estimate on-slope speed accurately is an important prerequisite for the reduction of accidents on ski slopes, and therefore prevention programs should include training of speed estimation, which could for example easily be integrated into snowboard education courses.

So far, individual estimation and feedback on speed were the only means for ski resort users to discern their speed-related risk-taking behaviours, but emerging GPS-based technologies are now providing more accessible means for them to become aware of their actual speed in real time. Resort managers may hope that the increasing use of technology such as fixed radars, photocells, dashboards, and other devices with GPS capabilities such as smart phones, watches or goggles, can be used as prompts to educate users regarding their speed, increasing their ability to estimate it and, in general, their understanding of their own behaviour [27].

Finally, a limitation of this study was that even though instructors assisting in the data gathering process stressed to participants that the research was focused on their usual riding of the slope, participants might have attempted to snowboard at higher speeds than their normal speeds because of the fact that their speeds were being measured. Additionally, our results are primarily generalizable to snowboarders visiting the Pyrenees, who are mostly Spanish and French. However, our results on actual maximum snowboard speeds seem to be consistent with results from the Alps and North America [15, 19].

## Conclusion

In conclusion, the factors considered in this study seem to affect both actual maximum speeds and the ability to estimate them in recreational adult snowboarders. Higher actual speed, female gender, lower skill, and an age over 50 years were associated with a decreased ability to estimate actual speed. Besides the importance of having the skills to manage speed and stay in control, which is paramount to avoid accidents on ski slopes [13], the ability to estimate actual speed accurately adds to accident and injury prevention because of being a relevant factor to ride within the limits of both indicated speed zones and protective equipment. Therefore, having snowboarders informed about the benefits of speed self-awareness is a key matter for prevention purposes.

## Supporting information

S1 Fig Plot. (TIF)

S1 Dataset Records. (XLSX)
