# Supplementary figures and images for: Injury prevention: Individual factors affecting adult recreational snowboarders’ actual and estimated speeds on regular slopes
Source: PLoS One. 2021 Feb 10;16(2):e0246931. doi: 10.1371/journal.pone.0246931 (PMC7875360; doi:10.1371/journal.pone.0246931)

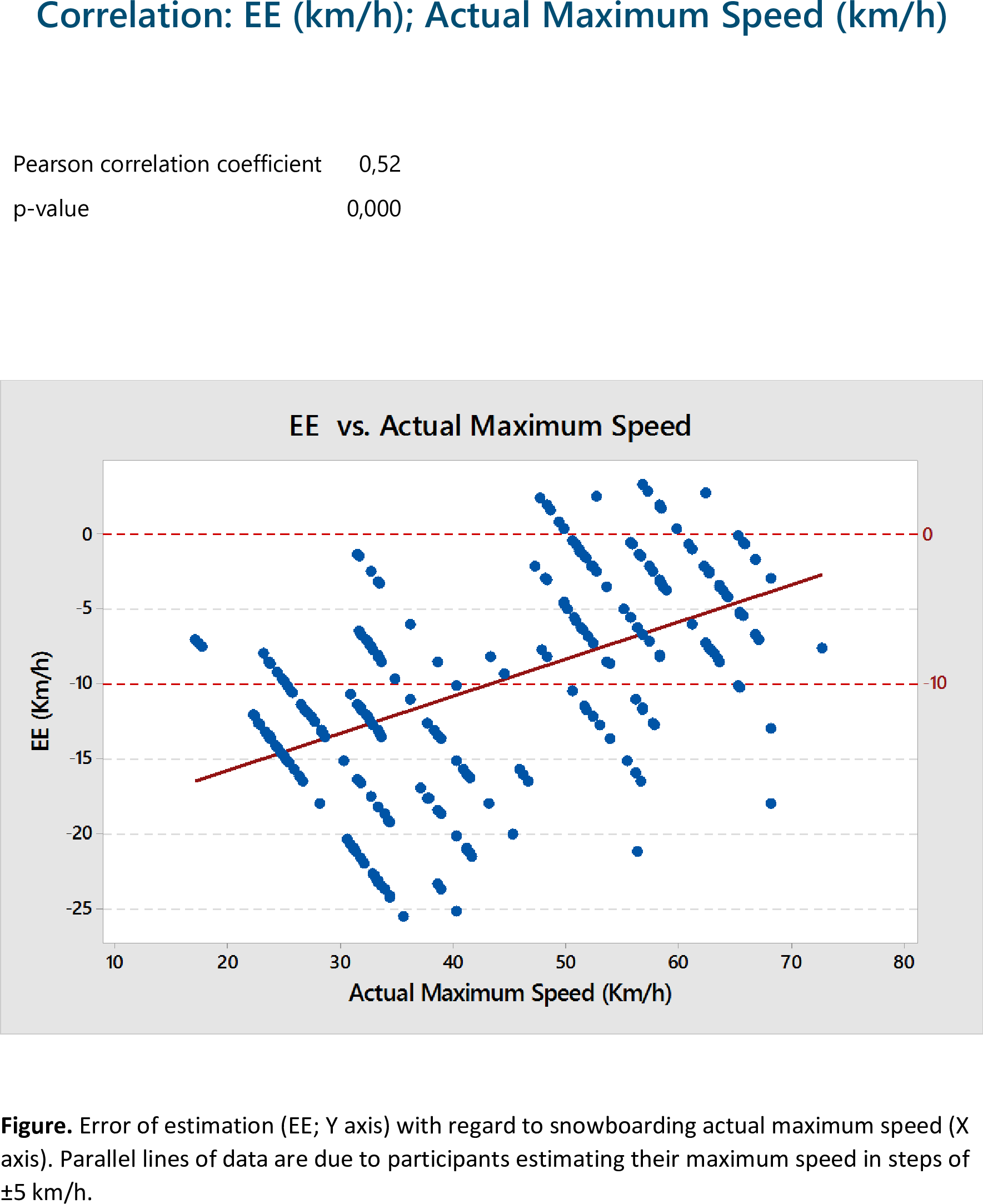

Supplement: S1 Fig — (TIF) [file pone.0246931.s001.tif]
